# Differences in Soil Bacterial Community Compositions in Paddy Fields under Organic and Conventional Farming Conditions

**DOI:** 10.1264/jsme2.ME18101

**Published:** 2019-02-13

**Authors:** Kazuki Suzuki, Manami Takemura, Takaaki Miki, Masanori Nonaka, Naoki Harada

**Affiliations:** 1 Institute for Research Promotion, Niigata University 2–8050 Ikarashi, Nishi-ku, Niigata 950–2181 Japan; 2 Graduate School of Science and Technology, Niigata University 2–8050 Ikarashi, Nishi-ku, Niigata 950–2181 Japan; 3 International Nature Farming Research Center 5632–1 Hata, Matsumoto, Nagano 390–1401 Japan; 4 Institute of Science and Technology, Niigata University 2–8050 Ikarashi, Nishi-ku, Niigata 950–2181 Japan

**Keywords:** MiSeq, organic rice soil, soil bacterial community

## Abstract

Soil bacterial community compositions and temporal changes in organic paddy fields were elucidated using a 16S rRNA gene amplicon sequencing analysis with a high-throughput next generation sequencer. At transplanting, bacterial community compositions in organic and conventional paddy fields were mostly similar despite differences in field management. The bacterial community composition in organic fields differed from that under conventional management during the rice growth period, possibly as a result of the decomposition process of organic fertilizers. However, differences in the frequency of tillage and photosynthetic bacterial inoculations in the organic plots had less of an impact on bacterial communities.

Organic agriculture refers to a farming system that markedly reduces the use of chemical fertilizers, pesticides, and genetically modified organisms, and aims to minimize any negative impact in order to improve long-term soil sustainability (8). Rice is now cultivated organically. Paddy soil is a group of anthropogenic soils with a long history of rice cultivation and is the most important farming system in Japan because rice is a staple food.

A more detailed understanding of soil bacterial ecology is very important for organic agriculture because diversity and community compositions influence the soil ecosystem, nutrient cycling, and agricultural production (17). Lopes *et al*. (12) compared the microbial diversity of organic and conventional paddy soils and revealed temporal variations in the organic field, but not in the conventional field using PCR-DGGE targeting the bacterial 16S rRNA gene. Although these analyses provided useful information, the techniques employed for the bacterial community analysis were insufficient to survey the full extent of microbial diversity.

Inoculations of commercial microbial materials containing photosynthetic bacterial strains are sometimes conducted in organic rice farming because organic farmers generally recognize that photosynthetic bacteria are beneficial in organic rice production. In addition to microbial inoculations, tillage management is regarded as an important practice for organic rice farming. In some organic rice fields, additional tillage is conducted after harvesting with the aim of promoting rice straw decomposition and controlling weeds (11). However, the effects of photosynthetic bacterial inoculations and the frequency of tillage on the soil bacterial community under organic rice farming have not yet been investigated in detail.

Rapid advances have been achieved in next generation sequencing technologies in recent years and offer new methods to examine soil microbial communities at high coverage and throughput. Lopes *et al*. (13) demonstrated that soil chemical properties and enzymatic activities correlated with variations in the bacterial community structure occurring in organic alfalfa-rice rotation paddy fields. However, the effects of organic management in paddy fields, including different frequencies of tillage and/or photosynthetic bacterial inoculations, on indigenous soil bacterial community compositions currently remain unclear.

In the present study, we investigated bacterial community compositions in organic and conventional paddy fields using a 16S rRNA gene amplicon sequence analysis with a next generation sequencer. The objectives of the present study were 1) to elucidate changes in soil bacterial communities in paddy fields under organic management and 2) to clarify the effects of the frequency of tillage and photosynthetic bacterial inoculations on bacterial communities.

Experimental plots (30 a each) were established in two adjacent rice fields located in Matsumoto, Nagano, Japan (N 36.2150, E137.8709) in 2014. The FAO Soil classification of the rice fields was Gleysol. One of the rice fields had been maintained under conventional management and the other under organic management for 6 years. The soil chemical properties of the experimental fields are shown in Table S1.

In the conventional field, chemical fertilizers were incorporated at 40 kg N ha^−1^, 96 kg P_2_O_5_ ha^−1^, and 56 kg K_2_O ha^−1^ on 13^th^ May 2014 based on the fertilization standards recommended by the Agricultural Administration Department of the Nagano Prefectural Government (1), followed by submerging and puddling. Rice seedlings were transplanted on 19^th^ and 20^th^ May. Topdressing of 40 kg-N ha^−1^ and 12 kg-K_2_O ha^−1^ was performed on 26^th^ July. Agrochemical treatments were conducted as follows: benfuracarb at transplanting, butachlor and pentoxazone on 30^th^ May, cyhalofop butyl, simetryn, benfuresate, and 4-(4-Chloro-2-methylphenoxy) butanoic acid in late June, cyhalofop butyl and bentazon in early July, and etofenprox and tricyclazole on 22^nd^ July. Mid-summer drainage was performed from 3^rd^ July to 16^th^ July.

The organic field was tilled on 9^th^ May and 300 kg ha^−1^ rice bran and 400 kg ha^−1^ rapeseed oil cake were incorporated according to local farming practices. Half of the field was also tilled in the autumn of the previous year (23^rd^ October 2013), which was defined as the double tillage treatment in the present study. After submerging and puddling, rice seedlings were transplanted on 23^rd^ May. Bokashi fertilizer, which was made by a 2-month fermentation of rice bran and rapeseed oil cake as the main materials, was topdressed at 100 kg ha^−1^ on 26^th^ May. Mechanical weed control was performed four times in June. Four different treatment plots (72 m^2^ each) were established in the organic field by the combination of different numbers of tillage (single or double tillage) and photosynthetic bacterial inoculations (*Rhodopseudomonas palustris* strain NBRC 100246) under a 2^2^ factorial design without replication. In the inoculated plots, *R. palustris* cultured in Hoshino medium (10) was washed with sterile water several times and the cultures dispersed to sterile water (1.6×10^9^ MPN mL^−1^) were then inoculated into nursery boxes of rice seedlings at 160 L ha^−1^ just before transplanting. Mid-summer drainage was performed from 21^st^ July to 1^st^ August. The details of these agricultural practices are shown in Table S2.

Fifty grams of soil samples were collected with a hand shovel from a depth of 0–15 cm at five points and mixed well just after transplanting (26^th^ May), 2–4 weeks before the mid-summer drainage (21^st^ June), and at the rice grain-filling stage (21^st^ August). Soil samples were kept at 4°C during transportation. After arriving at the laboratory, soil samples were homogenized together to produce a single plot sample and a proportion of each sample was stored at −80°C until soil DNA extraction.

Soil DNA was obtained from 0.5 g of homogenized soil samples using ISOIL for Beads Beating (Nippon Gene, Tokyo, Japan), according to the manufacturer’s instruction. The partial bacterial 16S rRNA gene (V4 region) was amplified using the bacterial primers 515F/806R with the linker sequences for Nextera primers (Illumina, San Diego, CA, USA). PCR products were purified using a High Pure PCR Product Purification Kit (Roche, Basel, Switzerland), and PCR barcode indexing was then performed with Nextera primers. Amplicons were purified with a QIAquick^®^ Gel Extraction Kit (Qiagen, Venlo, Netherlands) according to the manufacturer’s instructions. Amplicons were paired-end sequenced on an Illumina MiSeq platform (Illumina) at a read length of 2×300 bp. Sequencing was performed by Fasmac (Atsugi, Japan).

Sequencing data were processed and analyzed in QIIME 2 Core 2018.2 (3). Sequences were paired-end joined and filtered through a quality check and chimera check using DADA2 (2). A taxonomic analysis was performed using the q2–feature–classifier plugin trained on Greengenes 13_8 99% OTUs.

To compare bacterial communities among the samples, sequence read numbers were normalized to the minimum number of the sample by random subsampling. A cluster analysis and heat map analysis were performed based on the proportions of the bacterial community at the genus and species levels, respectively.

In a total of 745 OTUs were obtained at the genus level in the present study, with 97% having a <1% abundance ratio. An average of approximately 500 OTUs were obtained from each plot.

The proportions of the bacterial community at the phylum and order levels are shown in Fig. S1. In all treatments, the proportion of each phylum was generally as follows: approximately 25–30% *Proteobacteria*, 20% *Chloroflexi*, 15% *Acidobacteria*, and 10% *Actinobacteria* (Fig. S1a). At the order level, approximately 60% of the bacterial proportion was occupied by the top 20 orders. The dominant orders were as follows: approximately 7% *Rhodospirillales*, 6% *Rhizobiales*, 5% iii 1–15 (*Acidobacteria*–7), and 4% *Nitrospirales* (Fig. S1b). The proportion of OTUs related to the photosynthetic bacterial inoculum (*Bradyrhizobiaceae* sp.) was approximately 0.3%, and no significant difference was observed between the inoculated and non-inoculated fields at mid-summer drainage or in the grain-filling period (*P*>0.05, two-way ANOVA). Bacterial diversity indices (Shannon and Faith’s phylogenetic diversity) significantly increased during the cultivation period (*P*<0.05, Tukey-Kramer test) (Table S3), while no significant differences were observed among the treatments.

The cluster analysis based on the frequency of bacterial compositions at the genus level revealed differences in bacterial communities between conventional and organic farming (Fig. 1). At transplanting, bacterial community compositions were mostly similar among the plots despite differences in field management. After transplanting, bacterial community compositions, excluding OsP3, in the organic plots differentiated slightly more from those in the conventional plot (Fig. 1). There were no significant differences in rice yields among the treatments (see Table S4), indicating that differences in bacterial community compositions did not influence rice production yields in experimental fields in the present study.

Organic amendments have an impact on the soil bacterial community and changes community compositions under upland (4) and paddy conditions (5). Although our results were consistent with these findings, organic management had a negligible effect on bacterial community compositions at transplanting in the present study. A previous study reported that soil bacterial community compositions and diversity in paddy soil change temporally during the incubation period (15). Our results also revealed a temporal change in the bacterial community under organic management. Daquiado *et al*. (5) performed a pyrosequencing analysis of the soil bacterial 16S rRNA gene in long-term inorganic and compost fertilization in paddy fields. They found that organic fertilizer amendments activated diverse groups of Gram-positive microorganisms (*Firmicutes* and *Actinobacteria*), and *Rhizobiales*, which aid nutrient cycling, were influenced by the application of compost. In the present study, organic management significantly increased the relative abundance of the phylum *Firmicutes* (*P*<0.05, Tukey-Kramer test), but not the phylum *Actinobacteria* or order *Rhizobiales* (Fig. S1a and b).

After mid-summer drainage, bacterial community compositions under organic management slightly differed from the others (Fig. 1). Rui *et al*. (16) reported that the bacterial community composition shifted during plant residue decomposition in a soil incubation experiment under flood conditions, and this succession may have been related to resource availability. Our results indicated that organic management affected the bacterial community during the organic carbon decomposition process as a result of the development of soil reduction. Another possibility is the effect of topdressing of additional fertilizer during cultivation.

The community heat map and clustering of the top 20 dominant species were shown in Fig. 2. Based on the proportions of the top 20 dominant species, conventional and organic treatments were separated from each other with one exception, suggesting that the dominant species were affected by differences in fertilization management, while the frequency of tillage or photosynthetic bacterial inoculations had a weaker effect on the relative abundance of dominant species. Fig. 2 also showed that the top four dominant species (species 01, 02, 03, and 04) were clearly differentiated from the other 16 species on the cluster dendrogram. This result suggests that the four species have different sensitivities for the difference in management or growth period. However, it was difficult to discuss their ecological functions in the present study because only four of the 20 species were identified at the genus level (Table S5).

We examined the inoculation of *R. palustris* in the present study. Although the bacterial inoculation may have had an impact on soil bacterial community compositions (Fig. 1), the proportion of related OTUs was similar between the inoculated and non-inoculated fields. Harada *et al*. (9) reported that a *R. palustris* inoculation did not significantly affect a phototrophic purple bacteria population when rice straw was applied; however, this inoculation increased the rice grain yield. It has also been reported that the inoculation of plant growth-promoting bacteria did not have a significant impact on culturable microbial communities (6). Our results are consistent with these findings; however, further investigations are required to evaluate the impact of inoculations in more detail because inoculation methods may influence the fate of the inoculum.

Although tillage management may affect the bacterial community in cropland (14), the effects of the frequency of tillage have not been reported under paddy field conditions. Our results suggest that the frequency of tillage had a weaker effect on the bacterial community than fertilization management (Fig. 1).

In conclusion, organic management mainly affected bacterial community compositions after transplanting. During the rice growth period, gradual changes were observed in the bacterial community and the bacterial community under organic management differed from that under conventional management, possibly as a result of the decomposition process of organic fertilizers. On the other hand, the frequency of tillage and photosynthetic bacterial inoculations had a weaker effect on bacterial communities. The present results contribute to our understanding of the responses of bacterial communities to organic management in paddy fields.

## Supplementary Information



## Figures and Tables

**Fig. 1:**
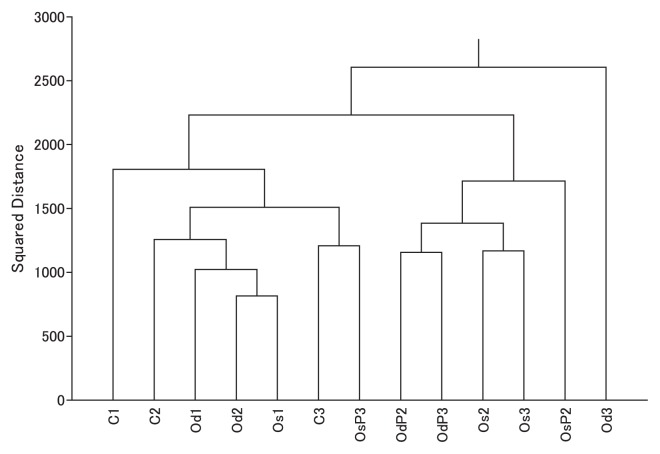
Cluster analysis of bacterial communities at the species level. C, conventional; Os, organic with single tillage; Od, organic with double tillage; P, photosynthetic bacteria inoculation. Sampling periods are shown as follows: 1, just after transplanting; 2, before mid-summer drainage; 3, grain-filling period.

**Fig. 2:**
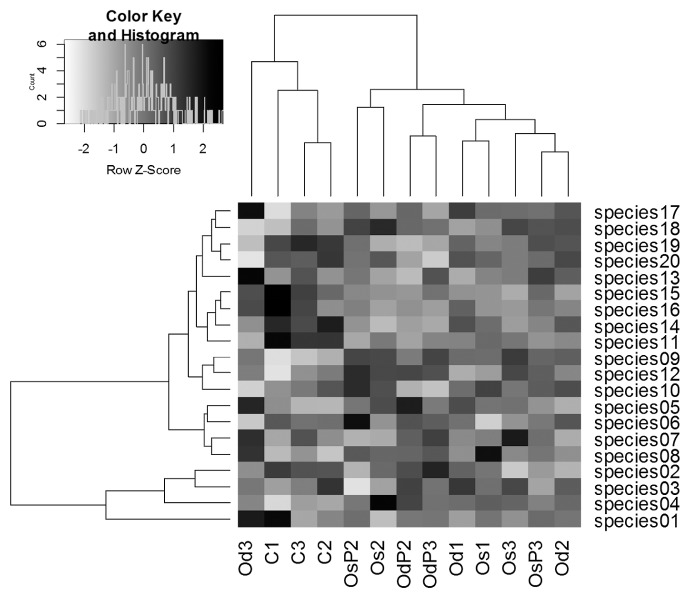
Heatmap and cluster analyses of top 20 dominant bacterial species. C, conventional; Os, organic with single tillage; Od, organic with double tillage; P, photosynthetic bacteria inoculation. Sampling periods are shown as follows: 1, just after transplanting; 2, before mid-summer drainage; 3, grain-filling period.
