# Supplementary material for: Differences in Soil Bacterial Community Compositions in Paddy Fields under Organic and Conventional Farming Conditions
Source: Microbes Environ. 2019 Feb 13;34(1):108–11. doi: 10.1264/jsme2.ME18101 (PMC6440725; doi:10.1264/jsme2.ME18101)
Supplement: Supplementary file 1 [file 34_108_s1.pdf]

Fig. S1. Frequency of top 10 dominant bacterial phyla (a) and top 20 dominant bacterial orders (b) detected in the present study. C, conventional; Os, organic with single tillage; Od, organic with double tillage; P, photosynthetic bacteria inoculation. Sampling periods are shown as follows: 1, just after transplanting; 2, before mid-summer drainage; 3, grain-filling period.

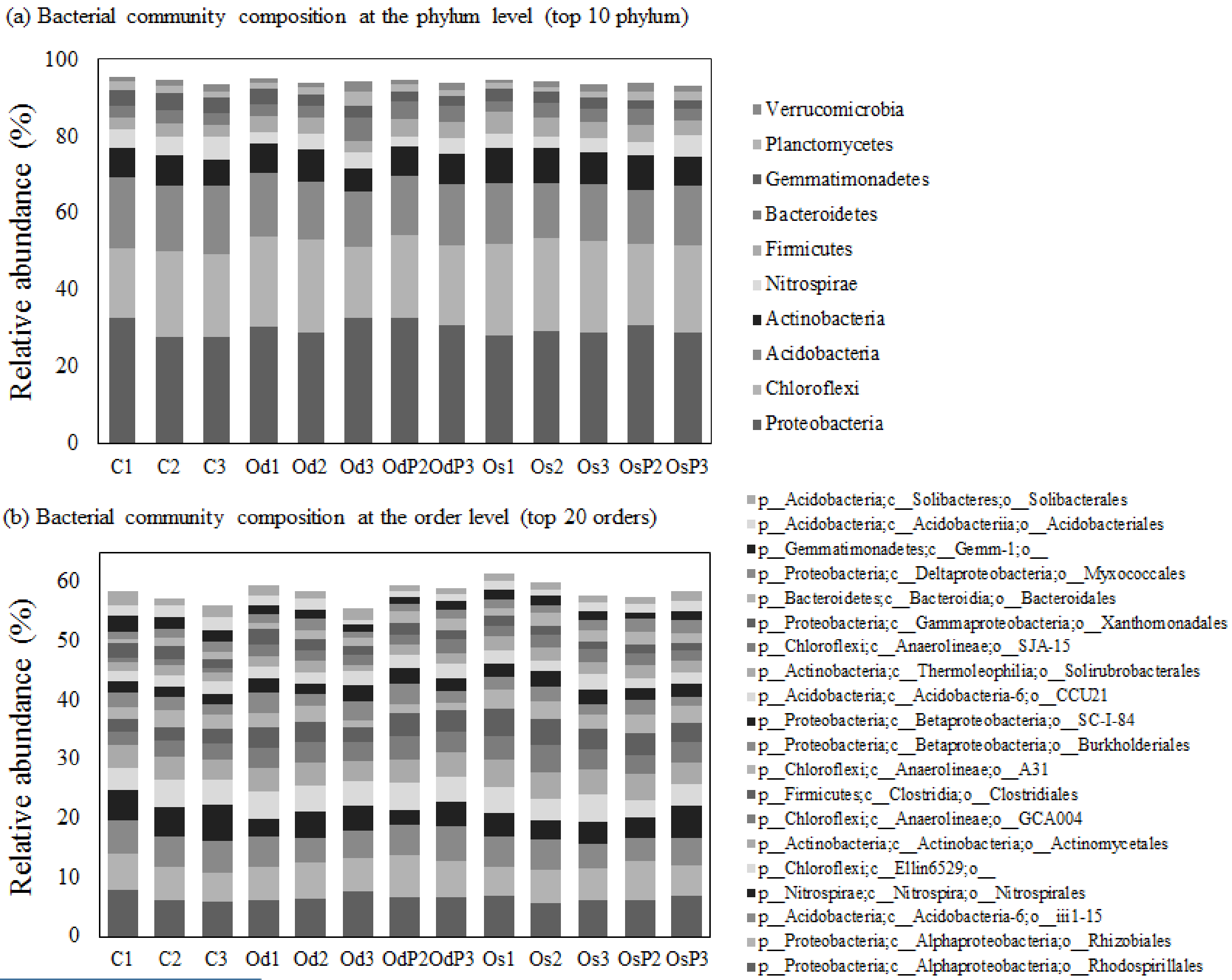

Fig. S1 Kazuki Suzuki

Table S1. Soil chemical properties in the experimental fields. AP: available P (P_2O_5) by Dickman and Bray's method (6); ExK: exchangeable K (K_2O); ExCa: exchangeable Ca (CaO); ExMg: exchangeable Mg (MgO).

Rice field	pH (H_2O)	EC (mS cm^{-2})	T-C (g-C kg^{-1})	T-N (g-N kg^{-1})	NH_4-N (mg kg^{-1})	NO_3-N (mg kg^{-1})	AP (mg kg^{-1})	ExK (mg kg^{-1})	ExCa (mg kg^{-1})	ExMg (mg kg^{-1})	CEC (cmol(+) kg^{-1})	Fe_2O_3 (g kg^{-1})
Conventional	5.53	0.03	26.1	2.5	7.3	2.9	449	141	1200	157	7.71	5.43
Organic	5.55	0.03	29.5	2.8	6.7	3.3	396	151	1090	126	8.67	7.80

Table S2. The detailed schedules of agricultural practices in this study. C: conventional management, O: organic management, s: single tillage, d: double tillage, P: photosynthetic bacteria inoculation; –: not performed, <: same as the left column

	Rice field				
Field management	C	Os	OsP	Od	OdP
Tillage					
- Autumn	Late Nov	–	–	23 Oct	<
- Spring	13 May	9 May	<	<	<
Submerging/puddling	14-19 May	15-20 May	<	<	<
Transplanting	19-20 May	23 May	<	<	<
PSB application	–	–	23 May	–	23 May
1st soil sampling	26 May	<	<	<	<
Organic application*	–	26 May	<	<	<
Application of agrochemicals	5 times in May-Jul	–	–	–	–
Weeding	–	4 times in Jun	<	<	<
2nd soil sampling	21 Jun	<	<	<	<
Middrainage	3-16 Jul	21 Jul-1 Aug	<	<	<
Topdressing	26 Jul	–	–	–	–
3rd soil sampling	21 Aug	<	<	<	<

* Bokashi fertilizer were produced by 2-months fermentation of 30 kg rice bran, 40 kg rapeseed oil cake, 2 kg rice husk, 1 kg eggshell, 0.3 L molasses and 30 L H_2O with lactic acid bacteria and yeast

Table S3. Bacterial Shannon-Wiener diversity and Faith's phylogenetic diversity (PD) based on species level relative abundance. Values with different characters indicate significant difference ($p < 0.05$). C: conventional management, O: organic management, s: single tillage, d: double tillage, P: photosynthetic bacteria inoculation.

	C	Od	OdP	Os	OsP	Average (±SD)			
Shannon-Wiener									
Transplanting	9.83	9.65		9.54		9.67	±	0.14	a
Middrainage	9.98	9.99	9.76	9.75	10.17	9.93	±	0.18	ab
Harvest	10.07	9.85	9.93	10.11	10.09	10.01	±	0.12	b
Faith's PD									
Transplanting	98.48	87.37		84.06		89.97	±	7.55	a
Middrainage	106.05	104.24	93.03	93.68	119.97	103.39	±	11.01	ab
Harvest	110.46	106.83	102.86	114.19	116.15	110.10	±	5.40	b

Table S4. Rice yields in the experimental plots.

Plot	Number of panicles (m^{-2})	Straw yield ($kg\ ha^{-1}$)	Grain yield ($kg\ ha^{-1}$)	Brown rice yield ($kg\ ha^{-1}$)	1000-grain weight (g)
Conventional (C)	360	5230	5945	4663	21.8
Organic					
Double tillage (Od)	417	5448	5653	4297	21.4
Double tillage +PSB (OdP)	422	5618	5832	4564	21.3
Single tillage (Os)	415	5409	5947	4635	21.4
Single tillage +PSB (OsP)	391	4746	4870	3792	21.2

+PSB; photosynthetic bacterial inoculation.

Table S5. Molecular taxonomic information of top 20 dominant species based on 16S rRNA gene. Species01 has highest and species20 has lowest relative abundance in top 20 dominant species.

	Phylum	Class	Order	Family	Genus	Species
species01	Proteobacteria	Alphaproteobacteria	Rhodospirillales	Rhodospirillaceae		
species02	Acidobacteria	Acidobacteria-6	iii1-15			
species03	Chloroflexi	Ellin6529				
species04	Chloroflexi	Anaerolineae	GCA004			
species05	Proteobacteria	Betaproteobacteria	SC-I-84			
species06	Firmicutes	Clostridia	Clostridiales	Clostridiaceae	*Clostridium*	
species07	Proteobacteria	Alphaproteobacteria	Rhizobiales	Hyphomicrobiaceae	*Rhodoplanes*	
species08	Acidobacteria	Acidobacteria-6	CCU21			
species09	Actinobacteria	Actinobacteria	Actinomycetales	Mycobacteriaceae	*Mycobacterium*	
species10	Chloroflexi	Anaerolineae	SJA-15			
species11	Chloroflexi	Anaerolineae	A31			
species12	Gemmatimonadetes	Gemm-1				
species13	Bacteroidetes	Bacteroidia	Bacteroidales			
species14	Nitrospirae	Nitrospira	Nitrospirales	FW	*4-29*	
species15	Acidobacteria	iii1-8	SJA-36			
species16	Proteobacteria	Betaproteobacteria				
species17	Acidobacteria	Solibacteres	Solibacterales			
species18	Chloroflexi	Anaerolineae	pLW-97			
species19	Chloroflexi	Anaerolineae	Anaerolineales	Anaerolinaceae		
species20	Acidobacteria	Acidobacteriia	Acidobacteriales	Koribacteraceae		
